# Visual atrophy rating scales and amyloid PET status in an Alzheimer's disease clinical cohort

**DOI:** 10.1002/acn3.51749

**Published:** 2023-03-05

**Authors:** Flavia Loreto, Anastassia Gontsarova, Gregory Scott, Neva Patel, Zarni Win, Christopher Carswell, Richard Perry, Paresh Malhotra

**Affiliations:** ^1^ Department of Brain Sciences Faculty of Medicine, Imperial College London London UK; ^2^ Department of Neuroradiology Imperial College Healthcare NHS Trust London UK; ^3^ UK Dementia Research Institute Care Research and Technology Centre Imperial College London and the University of Surrey London UK; ^4^ Department of Nuclear Medicine Imperial College Healthcare NHS Trust London UK; ^5^ Department of Neurology Imperial College Healthcare NHS Trust London UK

## Abstract

**Objectives:**

Visual rating scales (VRS) are the quantification method closest to the approach used in routine clinical practice to assess brain atrophy. Previous studies have suggested that the medial temporal atrophy (MTA) rating scale is a reliable diagnostic marker for AD, equivalent to volumetric quantification, while others propose a higher diagnostic utility for the Posterior Atrophy (PA) scale in early‐onset AD.

**Methods:**

Here, we reviewed 14 studies that assessed the diagnostic accuracy of PA and MTA, we explored the issue of cut‐off heterogeneity, and assessed 9 rating scales in a group of patients with biomarker‐confirmed diagnosis. A neuroradiologist blinded to all clinical information rated the MR images of 39 amyloid‐positive and 38 amyloid‐negative patients using 9 validated VRS assessing multiple brain regions. Automated volumetric analyses were performed on a subset of patients (*n* = 48) and on a group of cognitively normal individuals (*n* = 28).

**Results:**

No single VRS could differentiate amyloid‐positive from amyloid‐negative patients with other neurodegenerative conditions. 44% of amyloid‐positive patients were deemed to have age‐appropriate levels of MTA. In the amyloid‐positive group, 18% had no abnormal MTA or PA scores. These findings were substantially affected by cut‐off selection. Amyloid‐positive and amyloid‐negative patients had comparable hippocampal and parietal volumes, and MTA but not PA scores correlated with the respective volumetric measures.

**Interpretation:**

Consensus guidelines are needed before VRS can be recommended for use in the diagnostic workup of AD. Our data are suggestive of high intragroup variability and non‐superiority of volumetric quantification of atrophy over visual assessment.

## Introduction

All recent diagnostic criteria^1^, ^2^ recommend the use of MRI as the first step after clinical evaluation of suspected Alzheimer's Disease (AD) to examine patterns of grey matter atrophy, exclude brain lesions, and assess vascular white matter disease and microbleeds. Despite an increasing availability of in vivo biomarkers of AD neuropathology, MRI remains one of the most widely used and accessible diagnostic examinations for AD.^3^

In current clinical practice, the interpretation of MR images relies on visual assessment, with most reports consisting of a qualitative description of abnormal brain features. Several automated and semi‐automated quantification methods have been developed with the goal of making MRI assessment less subjective and more sensitive to neuroanatomical changes. However, visual rating scales (VRS) represent a simpler, more accessible approach to obtain quantitative measures of atrophy. These involve visual assessment of images and rating the degree of atrophy as an integer score on a Likert scale. The clinical implementation of VRS is relatively widespread compared to that of segmentation tools which, despite significant progress, are scarcely used in clinical practice due to limited resources, time constraints, and unclear reliability at the individual level.^3^, ^4^, ^5^ Moreover, most studies to date suggest diagnostic equivalence between MRI segmentation and VRS.^4^, ^6^

The most widely used and validated VRS for the assessment of the MTL is Scheltens and colleagues’ medial temporal atrophy (MTA) scale.^7^ This scale's diagnostic accuracy has been extensively investigated in AD, but findings are mixed. A recent meta‐analysis found pooled sensitivity and specificity, respectively, equal to 74% and 88% for its use in the differentiation of AD from healthy controls (HC) and concluded that MTA is a reliable diagnostic marker for AD.^8^ However, sensitivity and specificity varied widely across the reviewed studies, especially for the identification of AD from other forms of dementia, which is of relevance to clinical practice. In these cases, MTA sensitivity ranged between 50% and 89% and specificity between 11% and 94%.^8^ Heterogeneity among studies was attributed to differences in the MTA rating methods used.^8^ However, the use of different cut‐offs for the definition of rating scale abnormalities is also a major issue and source of heterogeneity that very few studies seem to have acknowledged and attempted to address so far.^9^, ^10^, ^11^, ^12^

In view of the association between age and atrophy, age‐specific VRS cut‐offs have been proposed with the aim of maximizing scale sensitivity and specificity by differentiating “age‐appropriate” atrophy from pathological atrophy. However, conservative cut‐offs increase specificity at the expense of sensitivity while more liberal cut‐offs present the converse problem. Different research teams have attempted to identify age‐adjusted cut‐offs that provide the best balance between the two, but these have often come to different conclusions. For example, at least four different sets of age‐specific cut‐offs have been proposed for the MTA to date^7^, ^9^, ^10^, ^11^, ^13^ (see Table S1). Other researchers, instead, have established different cut‐off scores according to clinical populations rather than age groups.^14^, ^15^ Moreover, studies have differed in the way they analyzed the left and right hemisphere scores, with some using the sum, others the highest score, while the majority incorporated the mean of both sides (see Table S2). Despite representing a major source of heterogeneity, the cut‐off issue seems to be incompletely acknowledged in the literature, to the point that some studies on VRS diagnostic accuracy have not reported the scores used to determine abnormality.^16^, ^17^

Evidence that 20–30% of AD patients, especially those with early onset (EOAD), show parietal‐dominant patterns of atrophy^18^, ^19^ led to the development of the posterior atrophy (PA) scale.^20^ In Table S2, we have reviewed the data extracted from 14 peer‐reviewed research articles found using PubMed as the main database (in July 2022). Studies were selected if these assessed the diagnostic and/or prognostic role of Koedam et al.'s Posterior Atrophy (PA) scale,^20^ Scheltens et al.'s MTA scale,^7^ and a variable range of other VRS, in AD patients. An exhaustive review of all available literature is beyond the scope of this study, but this overview provides insight into the role of PA and MTA, separately and in combination, in AD. Currently available evidence converges towards incremental diagnostic value of PA over MTA for the identification of EOAD from younger controls and other forms of dementia.^10^, ^14^, ^15^, ^19^, ^21^, ^22^

Besides the MTA and PA, other VRS for the assessment of orbitofrontal,^23^, ^24^, ^25^ fronto‐insular (FI), anterior cingulate (AC), and entorhinal^26^, ^27^ areas, have been developed for the identification of AD from other forms of dementia, such as frontotemporal dementia (FTD).^15^, ^28^ However, their implementation in research and clinical settings is limited thus far. In addition to atrophy measures, small vessel disease, which is typically seen as areas of white matter hyperintensity (WMH) on fluid‐attenuated inversion recovery (FLAIR) MR images, is often assessed using the Fazekas scales for the quantification of deep white matter hyperintensities (DWMH) and periventricular hyperintensity (PVH).^29^

Few studies have directly compared the diagnostic performance of multiple scales in the same cohort.^14^, ^15^, ^30^ Here, we employed a comprehensive set of VRS for the assessment of MRI images by an expert neuroradiologist blinded to all clinical information. The aims of the study were to (i) examine the patterns of atrophy as measured by expert interpretation of MRI using validated VRS in a biomarker‐confirmed clinical cohort, (ii) explore the clinical correlates of VRS scores in patients with Alzheimer's pathology, (iii) test the association between visual and automated quantification of regional atrophy, (iv) critically evaluate the impact of VRS cut‐off selection on research findings.

## Methods

### Subjects

One‐hundred consecutive patients seen at the Imperial Memory Clinic between 2013 and 2015 were reviewed and included in the present study if they met the following inclusion criteria: (i) they were referred to our Memory Clinic for the investigation of possible AD; (ii) the diagnostic workup included both MRI and amyloid PET; (iii) if performed externally, the MR images were made available to our Centre; (iv) decision to perform amyloid PET followed appropriate use criteria^31^ and was made by consensus within the multidisciplinary team.^32^, ^33^ If patients had more than one MRI scan, we selected the scan preceding amyloid PET. Where this preceded amyloid PET by more than 12 months, we selected the one following amyloid PET if this was available and performed within 6 months. A total of 77 patients, 39 amyloid‐positive (Aβ‐pos) and 38 amyloid‐negative (Aβ‐neg), met the inclusion criteria. In addition, the MR images of 28 cognitively normal (CN) individuals (mean age ± SD = 71.88 ± 5.99 years) scanned at ICHT for research purposes were acquired for volumetric analyses.

### MRI acquisition

Most patients (90%) were scanned at our Centre using a 1.5 T Siemens MAGNETOM Avanto (repetition time = 900 ms; echo time = 3.37 ms; 160 slices/slab, voxel size = 1 × 0.5 × 0.5 mm). Patients scanned at a different centre before referral (*n* = 8) were not excluded to ensure consecutive selection. All patients had at least a T1‐weighted image available plus a variable range of additional sequences, such as FLAIR and T2*/SWI, which were included for the assessment of WMH and cerebral microbleeds. All CN individuals were scanned at ICHT using a 3T Siemens MAGNETOM Verio (repetition time = 900 ms; echo time = 2.52 ms; 176 slices/slab, voxel size = 1 × 1 × 1 mm.) T1‐weighted images were extracted to perform volumetric analyses.

### MRI analysis

#### Visual rating protocol

Visual ratings of the complete dataset (*n* = 77) were performed by a single expert neuroradiologist (A.G.) blinded to all clinical and pathological information, except patient age at MR scanning. Images were rated in the native space in keeping with standard clinical reads using Research PACS version 12.1.5.1046 (Philips Vue PACS). Cerebral atrophy across seven brain regions as well as deep and periventricular WMH were rated using 9 validated rating scales (Table 1). To ensure consistency, an electronic proforma was created to record visual rating scores and publicly available reference images were used at the training stage and throughout the study. For more information on the reference images used in this study see Enkirch et al (2018) for the ERICA scale,^26^ Wardlaw and Ferguson's guide for the FAZEKAS scale (https://www.ed.ac.uk/sites/default/files/atoms/files/white_matter_rating_qualitative_final.pptx), and Harper et al.'s supplementary material for the remaining scales.^15^ For all atrophy scales, left and right hemisphere scores were averaged to obtain a single score. Moreover, the rater recorded whether other neuropathologies (e.g., strategic infarct, normal pressure hydrocephalus, demyelination) were visible on MR images.

**Table 1:** Description of visual rating scales.

Scale	Brain regions/features	Plane‐modality	Score range
Orbitofrontal (OF)^15^, ^24^	Corpus callosum, width of the olfactory sulcus	*Coronal‐T1*	0–3
Anterior cingulate (AC)^15^, ^25^	Corpus callosum, width of the cingulate sulcus	*Coronal‐T1*	0–3
Frontoinsula (FI)^15^, ^24^	Width of the circular insular sulcus, anterior commissure	*Coronal‐T1*	0–3
Anterior temporal (AT)^15^, ^24^	Width of the anterior temporal sulci, aspects of the temporal pole	*Coronal‐T1*	0–4
Medial temporal atrophy (MTA)^7^	Width of the choroid fissure, width of the temporal horn, height of the hippocampus	*Coronal‐T1*	0–4
Entorhinal cortex (ERICA)^26^	volume of the entorhinal cortex and parahippocampal gyrus, width of the collateral sulcus, width of cleft between the entorhinal cortex and the cerebellar tentorium	*Coronal‐T1*	0–3
Posterior atrophy (PA)^20^	Width of the posterior cingulate and parieto‐occipital sulci, volume of the parietal lobes, and precuneus	*Coronal, axial, sagittal‐T1*	0–3
Fazekas^29^	areas of abnormal high signal intensity around the ventricles (periventricular, PVH) and in the deep white matter (DWMH)	*Axial‐FLAIR*	0–3

#### Volumetric analyses

To explore the relationship of MTA and PA scores with the respective volumes, volumetric analyses were performed on a subset of patients (*n* = 48) whose T1 images met FreeSurfer requirements, as well as on the CN group (*n* = 28). The *recon‐all* function of FreeSurfer 6.0 was used to extract estimates of brain volumes (https://surfer.nmr.mgh.harvard.edu/)^34^: hippocampal volumes (HV) were obtained from subcortical output files extracted through aseg.stats; parietal volumes were obtained from aparc.stats files using the Desikan atlas.^35^ Within the Desikan atlas, the parietal lobe is segmented across 5 regions in each hemisphere, namely the postcentral gyrus, the supramarginal gyrus, the superior and inferior parietal cortex, and the precuneus.^35^ The “mean parietal area” was obtained by averaging these 5 regions, separately for the left and right hemispheres. Recon‐all logs and images were inspected for major segmentation errors.

### Amyloid PET image acquisition and clinical reads

All 77 patients included in this study were scanned using a Siemens Biograph 64 PET/CT scanner. The amyloid PET procedure and clinical interpretation were performed as described elsewhere.^36^, ^37^ Briefly, images were acquired following post‐injection interval of intravenous 18F‐Florbetapir or 18F‐Florbetaben and were visually read as positive or negative by an experienced nuclear medicine radiologist. Equivocal cases were independently read by two nuclear medicine experts and by a third reader when there was disagreement.

### Clinical measures and diagnostic categorization

Clinical notes were retrospectively reviewed to determine the final clinical diagnosis, the syndromic stage at the time of MRI, the duration of cognitive impairment at the time of presentation to our Centre, and the age of onset of cognitive symptoms. For the Aβ‐pos group, we also recorded whether clinical presentation was characterized by amnestic or non‐amnestic symptoms. The Aβ‐neg group was further divided into “progressive Aβ‐neg” or “stable Aβ‐neg” based on the most likely cause of cognitive impairment.^36^ Patients were classified as “progressive” if they showed evidence of symptom progression over follow‐ups that was suggestive of a nonAD neurodegenerative process or if they had concomitant neurological conditions affecting brain integrity. Patients were defined as “stable” if the course of cognitive symptoms and the results of clinical investigations were suggestive of a non‐neurodegenerative cause of cognitive symptoms.

### Score dichotomization

VRS scores were dichotomized into “normal” or “abnormal” using age‐adjusted cut‐offs, with “normal” scores indicating age‐appropriate levels of atrophy. Where possible, we used validated VRS cut‐offs based on published data.^10^, ^13^ However, for the OF, AC, AT, FI, and ERICA, cut‐offs had to be established based on agreement between the clinical and research teams due to the lack of existing recommendations (Table S3).

### Statistical analysis

Demographic information was compared between groups using Student's t‐test for continuous data and *χ* ^2^ test for categorical data.

#### Visual assessment

**Figure 1:**
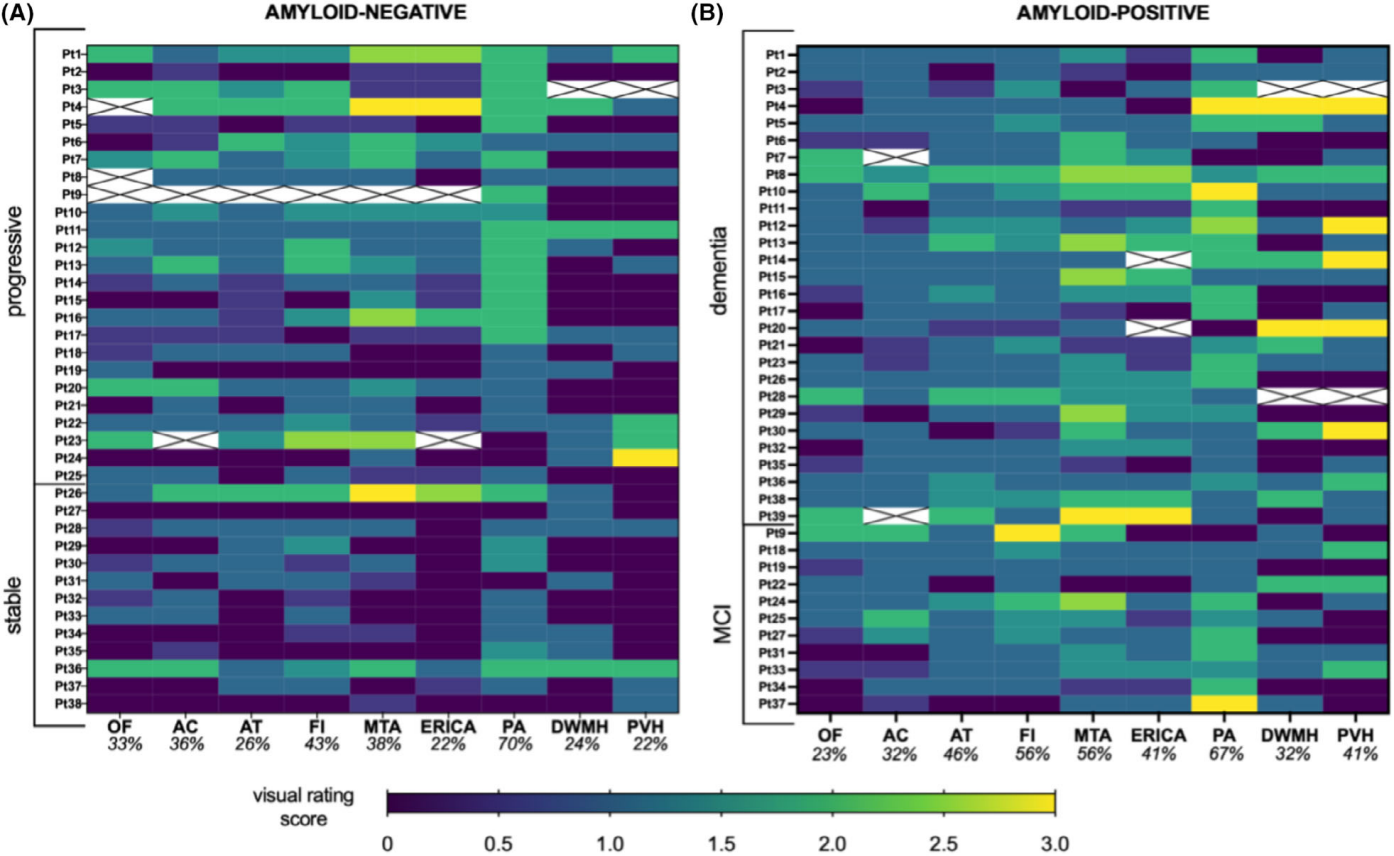
Heatmaps showing individual‐level visual rating scores in the amyloid‐negative (A) and amyloid‐positive (B) groups. Crossed‐out cells indicate missing visual rating score for that patient due to unavailable MR sequences. Percentages indicate the proportion of abnormal scores in each scale, dichotomized as per Table S3.

Higher visual rating scores indicate worse levels of atrophy. In this study, and in line with previous work, the left (LH) and right (RH) hemispheres scores were averaged to obtain a single score. Missing values, due to unavailable MR sequences or low‐quality images (Fig. 1), were not imputed. The distribution of VRS scores was tested through Shapiro–Wilk test, and group differences between the Aβ‐pos, stable Aβ‐neg, and progressive Aβ‐neg groups in non‐normally distributed data were analyzed using Kruskal‐Wallis nonparametric test and Mann–Whitney *U* for post hoc analyses. Separate Mann–Whitney *U* tests were performed on the amyloid‐positive and amyloid‐negative groups to test for the association between amyloid PET status and VRS score, irrespective of clinical group. In the Aβ‐pos group, we used Mann–Whitney *U* to test whether MTA and PA status (normal *versus* abnormal) was associated with age at the time of MRI, age of onset, duration (months) of cognitive impairment, while *χ* ^2^ tests were used to assess the association with sex and phenotype (amnestic *versus* non‐amnestic). These two regions were selected here to explore possible predictors of more (i.e., MTA) or less (i.e., PA) typical patterns of atrophy in this clinical population. The association between categorical variables was tested through *χ* ^2^ tests. In all post hoc analyses, *p*‐values were adjusted for multiple comparisons using Bonferroni correction.

#### Visual and quantitative assessment

The relationship between visual and quantitative measures of MTL and parietal volumes was tested separately for the left and right hemispheres (*n* = 48:22 Aβ‐pos, 26 Aβ‐neg). Partial correlation analyses, controlling for age, sex, and estimated total intracranial volume (eTIV), were used to investigate the association between HV and MTA scores and between mean parietal area and PA scores. In addition, multiple correlation analyses were run to test the association between left and right PA scores and each of the 5 lateralized Desikan parietal regions separately. The mean left and right HVs were compared between the Aβ‐pos (*n* = 22), Aβ‐neg (*n* = 26) and CN (*n* = 28) using an ANCOVA with age, sex, and eTIV as covariates. The left and right parietal areas were compared between the three groups using an ANCOVA with age, sex, and eTIV as covariates. Post hoc comparisons were performed on significant *p*‐values.

## Results

### Demographics

Clinical and demographic information is provided in Table 2. Aβ‐pos and Aβ‐neg patients did not significantly differ for age (*t*_(75)_ = 0.58, *p* = 0.56) or sex (*χ* ^2^_(1)_ = 0.11, *p* = 0.74) (Table 2).

**Table 2:** Demographic and clinical information according to amyloid PET status.

	Aβ‐pos (*n* = 39)	Aβ‐neg (*n* = 38)
*Demographics*		
Mean age ± SD (years)	67.19 ± 7.84	66.14 ± 8.14
Age range	49.4–81.1	53.8–87.9
Sex (%female)	41.02%	44.74%
*Clinical information*		
Duration of CI (months)	34.34 ± 25.67^1^	40.76 ± 31.92^2^
Median interval between MRI and amyloid PET (days)	148	210
%Early age of onset (<65 years)	51%	45%
Clinical diagnosis, *n* (%)	Amnestic AD, 28(72%) Nonamnestic AD, 11(28%)^3^	Stable MCI, 12(32%) Other dementia, 10(26%)^4^ Other neurological condition, 5(13%)^5^ Progressive MCI, 11(29%)

AD, Alzheimer's disease; Aβ‐neg, amyloid‐negative; Aβ‐pos, amyloid‐positive; CI, cognitive impairment; DLB, dementia with Lewy bodies; FTD, frontotemporal dementia; MCI, mild cognitive impairment.

^1^ Available for 38/39 patients.

^2^ Available for 33/38 patients.

^3^ Nonamnestic AD: visuospatial *n* = 5, language *n* = 5, behavioral *n* = 1.

^4^ Other dementia: dementia with Lewy bodies *n* = 3, frontotemporal dementia *n* = 7.

^5^ Other neurological conditions: normal pressure hydrocephalus *n* = 2, temporal lobe epilepsy *n* = 2, multiple cavernomas *n* = 1.

### Group comparisons

All Aβ‐pos patients received a clinical diagnosis of AD in line with National Institute on Ageing and Alzheimer's Association (NIA‐AA).^1^ Aβ‐neg patients, instead, formed a heterogeneous group, which we subdivided into “stable Aβ‐neg” (*n* = 12) and “progressive Aβ‐neg” (n = 26) (Table 2), as described above. Nonparametric testing on the three groups (i.e., Aβ‐pos, stable Aβ‐neg, and progressive Aβ‐neg) highlighted significant differences in the AC (Kruskal‐Wallis H_(2)_ = 6.08, *p* = 0.048), AT (Kruskal‐Wallis H_(2)_ = 7.66, *p* = 0.02), FI (Kruskal‐Wallis H_(2)_ = 6.24, *p* = 0.044), MTA (Kruskal‐Wallis H_(2)_ = 13.1, *p* = 0.001), ERICA (Kruskal‐Wallis H_(2)_ = 16.46, *p* < 0.001), PA (Kruskal‐Wallis H_(2)_ = 6.73, *p* = 0.03) scales, with the PVH approaching significance (Kruskal‐Wallis H_(2)_ = 5.72, *p* = 0.057). Bonferroni‐corrected Mann–Whitney *U* tests for each pairwise comparison revealed significantly higher scores in the Aβ‐pos group than the stable Aβ‐neg group in the AT (*p* = 0.01), FI (*p* = 0.02), MTA (*p* = 0.001), and ERICA (*p* < 0.001), with PA differences approaching significance (*p* = 0.054). The progressive Aβ‐neg group, instead, had significantly higher scores than the stable Aβ‐neg group in the MTA (*p* = 0.001), ERICA (*p* = 0.006), and PA (*p* = 0.045) scales. None of the scales differentiated the Aβ‐pos from the progressive Aβ‐neg group (lowest *p*‐value = 0.96, for the PVH scale). Heatmaps in Figure 1 illustrate the individual‐level patterns of scores in the three groups. Mann–Whitney *U* tests revealed significantly higher AT (*U* = 522.5, *p* = 0.025), MTA (*U* = 527.5, *p* = 0.04), ERICA (*U* = 419.5, *p* = 0.005), and PVH (*U* = 489.5, *p* = 0.023) scores in the amyloid‐positive group. In Figure S1 the mean rating scores of the current study population are plotted against those extracted from previous studies^14^, ^15^ for direct comparison.

#### Posterior and medial temporal atrophy: visual assessment

**Figure 2:**
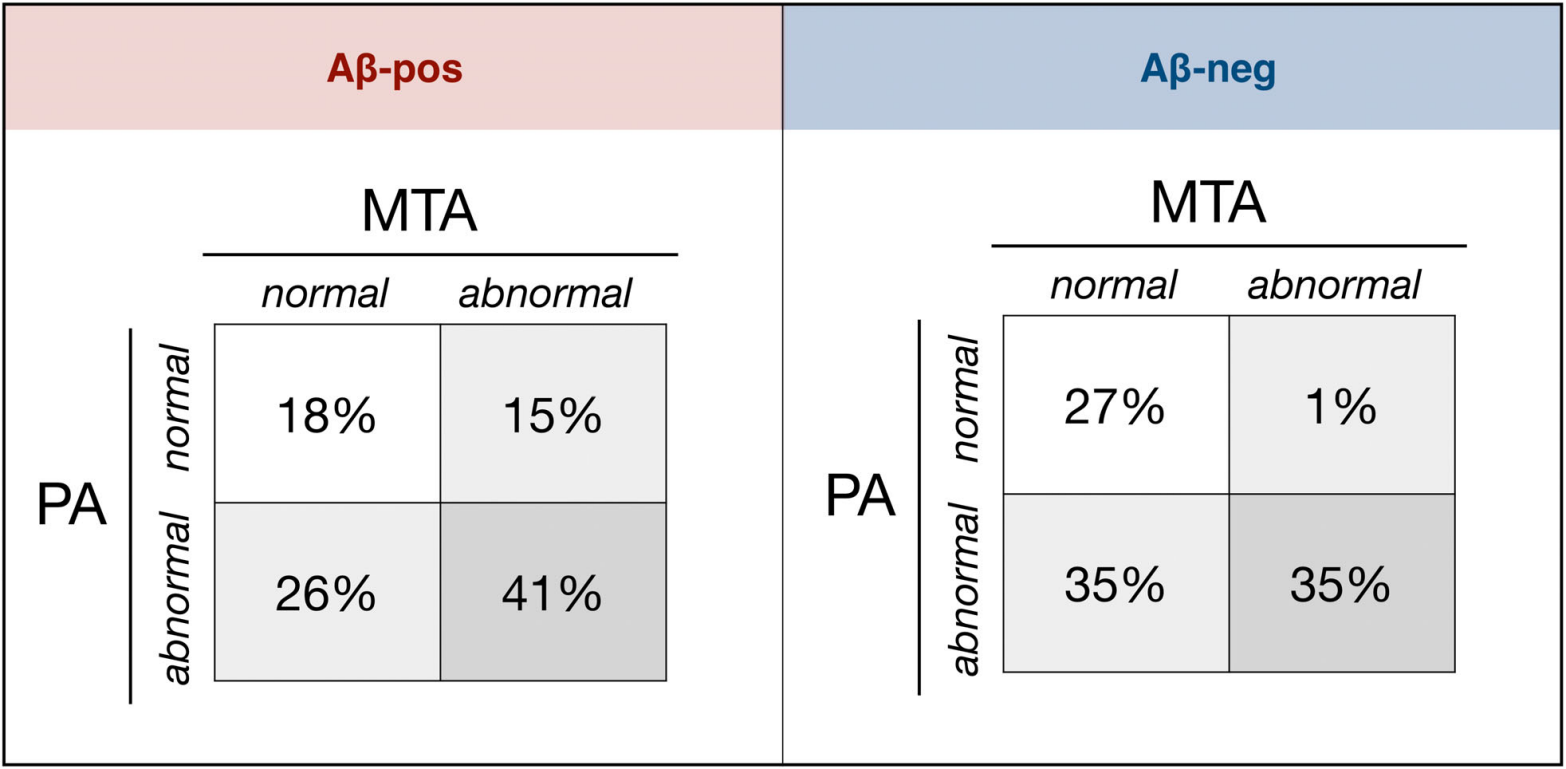
Combination of MTA and PA status in the amyloid‐positive and amyloid‐negative groups.

MTA and PA scores were dichotomized according to Rhodius‐Meester et al.'s^10^ age‐adjusted cut‐offs (see Table S1). Findings are shown in Figure 2. Aβ‐pos patients with normal MTA were comparable to those with abnormal MTA in all clinical and demographic variables except for age at the time of MRI, which was significantly lower in patients with abnormal MTA scores (normal: 67.1 ± 6.71 years, abnormal: 62.17 ± 8.91 years; *U* = 105 *p* = 0.02). Aβ‐pos patients with abnormal PA score were significantly younger at the time of MRI scanning (normal: 71.8 ± 5.61 years, abnormal: 64.9 ± 7.87 years; *U* = 78 *p* = 0.006) as well as at the time of symptom onset (normal: 69.87 ± 5.8, abnormal: 61.3 ± 8; *U* = 58 *p* = 0.001). Aβ‐pos patients with abnormal PA had also longer duration of cognitive symptoms at the time of their first memory clinic visit (normal: 23.1 ± 17.4 months, abnormal: 40.2 ± 27.6 months; *U* = 95 *p* = 0.038) and presented more frequently with non‐amnestic symptoms compared to those with normal PA scores (% non‐amnestic: normal 7.7%, abnormal 38.5%; *χ* ^2^_(1)_ = 4.05, *p* = 0.04).

#### Posterior and medial temporal atrophy: quantitative assessment

There was a significant negative association between left and right MTA scores and the respective hippocampal volumes (HV) (LH: *r* = −0.41 *p* = 0.006; RH: *r* = −0.47 *p* = 0.001) after controlling for age, sex, and eTIV. Mean left HV was 15.2% lower in subjects with left MTA scores ≥2 compared to those with normal MTA scores (3631mm^3^ vs 3079mm^3^). Mean right HV was 14.6% lower in subjects with right MTA scores ≥2 compared to those with normal MTA scores (3757 mm^3^ vs 3209 mm^3^). On the other hand, the left and right PA scores did not show a significant association with the mean parietal area (LH: *r* = −0.28 *p* = 0.06; RH: *r* = −0.21, *p* = 0.17) and, similarly, no significant correlations were found between PA scores and each parietal region separately.

**Figure 3:**
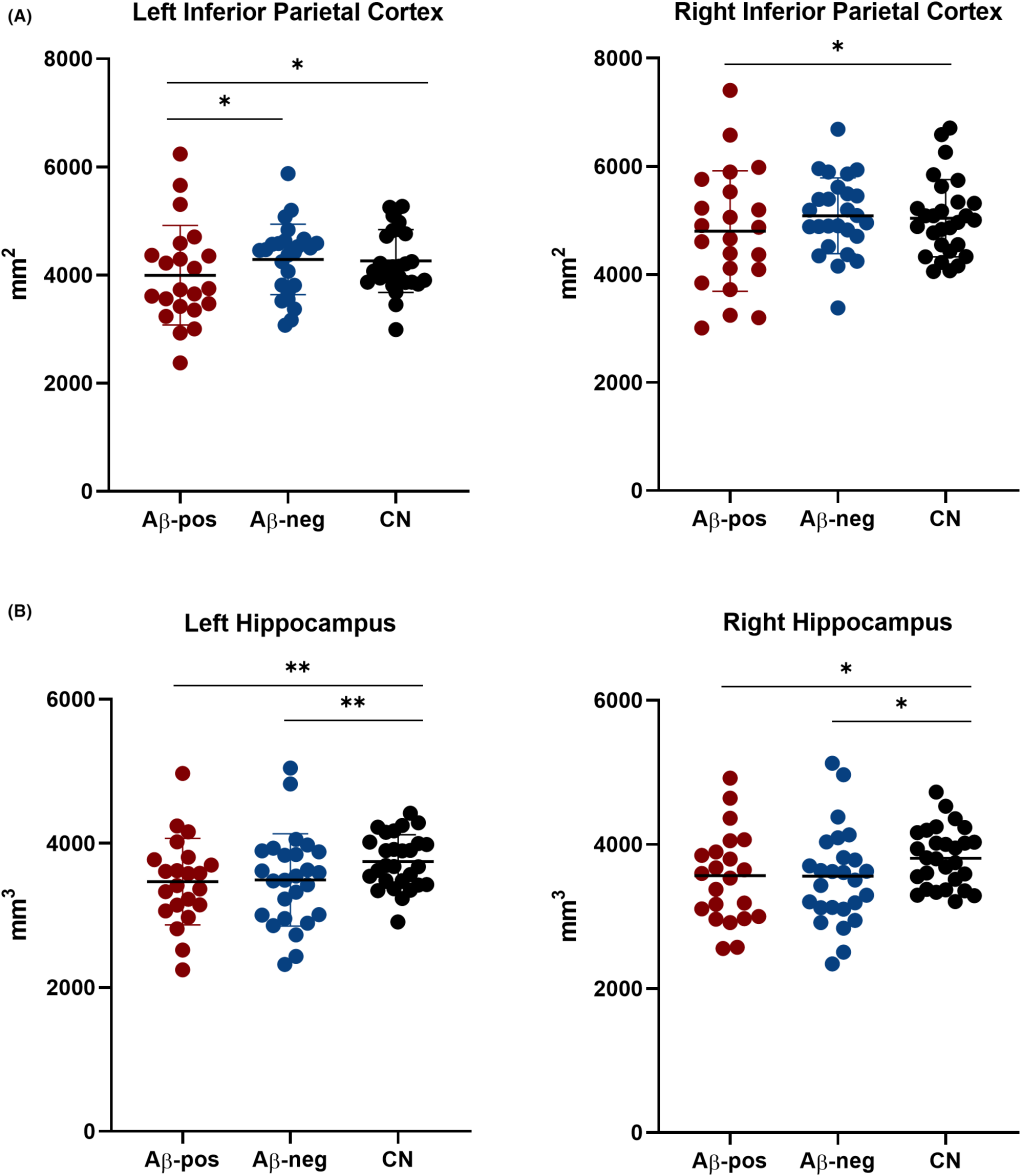
Quantification of the left and right parietal (A) and hippocampal (B) volumes in the amyloid‐positive, amyloid‐negative, and cognitively normal groups. ANCOVAs with “Group” as independent variable, and age, sex, and eTIV as covariates, revealed significant group differences for the hippocampal but not the parietal volumes. *p < 0.05; **p < 0.01; ns, nonsignificant.

The left (*F*_(2,70)_ = 4.85, *p* = 0.011) and right (F_(2,70)_ = 4.51, *p* = 0.014) inferior parietal cortices were the only parietal regions that significantly differed between groups, with smaller left inferior parietal cortex in the Aβ‐pos group than both the Aβ‐neg (LH: *p* = 0.034) and controls (LH: *p* = 0.019) groups, and smaller right inferior parietal cortex in the Aβ‐pos group than controls (RH: *p* = 0.018) (Fig. 3A). There were significant differences in both the left (*F*_(2,70)_ = 7.83, *p* = 0.001) and right (*F*_(2,70)_ = 5.33, *p* = 0.007) hippocampi (Fig. 3B), which were due to smaller volumes in the Aβ‐pos (LH: *p* = 0.002, RH: *p* = 0.02) and the Aβ‐neg (LH: *p* = 0.004, RH: *p* = 0.02) groups compared to controls; Aβ‐pos and Aβ‐neg groups did not significantly differ (*p* = 1.00).

### Alzheimer's disease group's characterization

**Figure 4:**
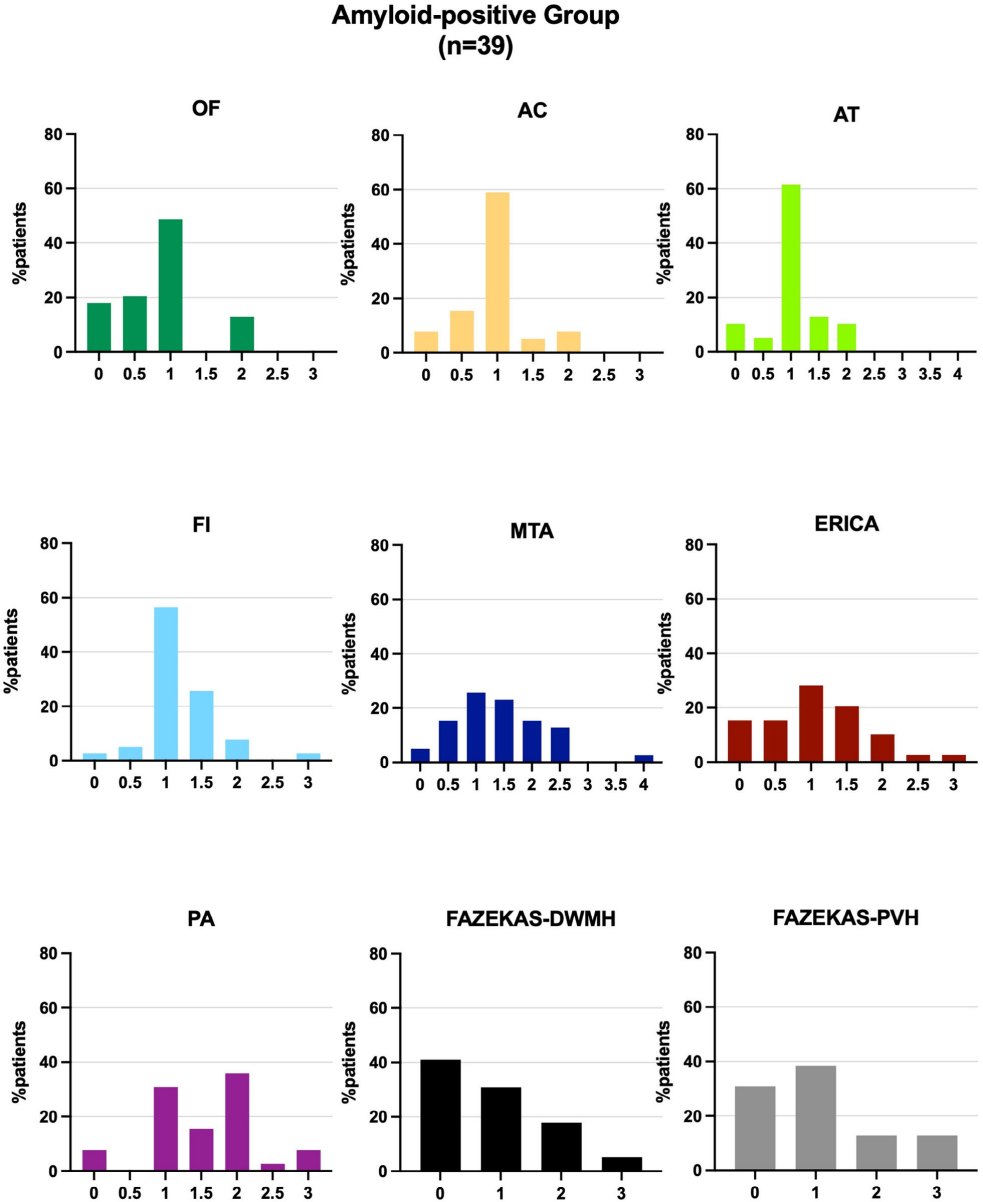
Distribution of visual rating scores for each rating scale. Plotted scores are the result of the average between the left and right hemisphere scores.

The individual VRS score profiles varied widely within the Aβ‐pos group (Fig. 1B), despite all patients having a clinical diagnosis of AD. Individual‐level number of abnormal scores ranged between 0 and 7. Notably, only 7.7% of AD patients had all scale scores in the abnormal range while 13% had no abnormal scores, indicating “age‐appropriate” atrophy across all cortical regions. Within the Aβ‐pos patients, the MTA and PA scales were the ones with the highest median score (median = 1.5, IQR = 1 for both) (Fig. 4). Abnormal scores on either the MTA or PA correctly identified 82% Aβ‐pos patients; however, abnormality in any of these two scales was also seen in 73% Aβ‐neg (stable and progressive) patients. With reference to the remaining scales, the Aβ‐pos group showed high proportions of abnormal scores in the FI (56%) and the AT (46%) scales, while the lowest proportion was seen in the OF scale (23%) (Fig. 1B).

#### The impact of cut‐off selection

It is important to note that we had to establish operational cut‐off values for the OF, AC, AT, FI, and ERICA scales due to the unavailability of validated age‐adjusted norms. Changing the cut‐off value from 1.5 to 2 for the 65–74 Aβ‐pos age group (in line with PA cut‐offs) for these scales would lead to a reduction of abnormal ERICA scores in the Aβ‐pos group from 41% to 35%, FI scores from 56% to 44%, AT scores from 46% to 41%, and AC scores from 32% to 30%, while abnormal OF scores would remain unchanged. Moreover, even where validated cut‐offs exist, these vary between studies. Replacing Rhodius‐Meester et al.'s^10^ MTA and PA cut‐off values with those suggested by Ferreira and colleagues^11^ (Table S1) in the Aβ‐pos group would lead to a reduction in MTA abnormality (from 56% to 49%) and a striking increase in PA abnormality (from 67% to 92%). This would corroborate prominent involvement of the posterior regions of the brain in our Aβ‐pos group as well as higher sensitivity of the PA scale. However, a similarly high proportion of Aβ‐neg patients would be classified as abnormal (86%) using these cut‐offs.

### Clinical correlates of Alzheimer's disease

OF scores were significantly higher in the amnestic group (*n* = 26) (*U* = 71, *p* = 0.005), while PA (*U* = 92.5, *p* = 0.045) and PVH (*U* = 84.5, *p* = 0.04) scores were significantly higher in the non‐amnestic group (*n* = 11) (Fig. S2). Mean MTA score was higher in the amnestic (mean ± SD = 1.52 ± 0.9) than the non‐amnestic (mean ± SD = 1.14 ± 0.5) group, but this did not reach significance (*p* = 0.197). Syndromic stage was not associated with VRS scores (*p* between 0.11 and 0.99). About half (49%) of Aβ‐pos patients had deep and/or periventricular WMHs, compared with 35% in the Aβ‐neg group, and their presence was not associated with atrophy scores (Aβ‐pos: *p* between 0.21 and 1, Aβ‐neg: *p* between 0.062 and 0.775). Notably, 31% of Alzheimer‘s disease patients had findings suggestive of other pathology including previous cerebral infarction, small vessel disease, and previous brain injury.

## DISCUSSION

In this study, we examined atrophy on structural MRI, as measured by expert visual assessment, in a biomarker‐confirmed clinical cohort of patients with suspected Alzheimer's disease.

The amyloid‐positive group had worse scores than the stable amyloid‐negative group in the MTA, PA, ERICA, AT, FI, but not the OF scale, whereas the progressive amyloid‐negative group had worse MTA, ERICA, and PA mean scores than the stable amyloid‐negative group. No single VRS differentiated amyloid‐positive from progressive amyloid‐negative patients. This is in line with previous studies finding lower diagnostic utility of a single brain region when comparing AD to other forms of dementia, rather than to controls,^14^ due to limited specificity.^38^, ^39^ Furthermore, AD may frequently present with extra‐temporal atrophy and relative sparing of the MTL.^40^, ^41^ In our clinical cohort, 44% of amyloid‐positive patients were deemed to have age‐appropriate levels of MTL atrophy. Unexpectedly, patients with a normal MTA score were on average older than those with abnormal MTA. This is in contrast with most available literature showing that hippocampal‐sparing forms of AD are associated with younger onset.^41^ There are two possible explanations for these findings: firstly, patients who are referred to amyloid‐PET scanning tend to present with atypical clinical features leading to diagnostic uncertainty and consequent need for biomarker examination, and atypically young age and inconclusive findings on standard diagnostic investigations are among those features.^31^, ^33^ It is, therefore, possible that patients who present with a typical Alzheimer‐like pattern of neurodegeneration, with involvement of the MTL, may be referred for amyloid PET on the basis of atypically young age in order to increase diagnostic confidence. Secondly, individuals presenting with atypical symptoms may take longer to be referred to a specialist clinic,^42^ meaning that they may be older and at more advanced disease stages by the time of amyloid PET referral.

We reviewed 14 research studies that assessed the performance of the MTA and PA scales, converging towards a possible incremental diagnostic value of PA in EOAD. In our amyloid‐positive group, patients with abnormal PA were indeed younger, had earlier age of onset and presented more often with non‐amnestic symptoms and longer disease duration compared to those with normal PA scores. The proportion of patients with a combination of abnormal PA and normal MTA scores (26%) was comparable to that found by previous studies of similar patients, while the proportion of those with abnormal MTA and PA (41%) was higher.^10^, ^19^ With reference to the remaining scales, the FI and the AT were the only two atrophy scales with over 40% of amyloid‐positive subjects having an abnormal score. The FI was also found to be promising in Harper et al.'s study, in which this was the best single scale, together with PA, for the identification of EOAD.^15^ The AT scale, instead, was found by previous studies to be better suited for the differentiation of other clinical groups, such as FTD and DLB.^14^, ^15^ The lowest proportion of abnormal scores and the lack of significant group differences in the OF scale provide indirect support for the specificity of this scale for the detection of other forms of dementia, such as FTD.^15^, ^28^ Notably, about half (49%) of amyloid‐positive patients had evidence of periventricular and/or deep WMH, despite their relatively young age, and this proportion was higher than that seen in the amyloid‐negative group (35%). Furthermore, the analysis of group differences according to amyloid PET status revealed significantly higher PVH scores in the amyloid‐positive than in the amyloid‐negative group. These findings are not in line with previous studies^10^ and would suggest WMH as a possible imaging feature of this specific clinical population.

The lack of association between hippocampal volumes and amyloid PET status is in keeping with previous studies suggesting non‐superiority of volumetric quantification of the MTL over visual assessment.^4^, ^6^ The extent of overlap of hippocampal volumes in patients with and without Alzheimer's pathology was striking and reinforces the need to rethink the role of this measure in AD diagnosis, especially in younger and atypical patients,^22^ who are frequently referred for amyloid PET^31^.^33^ Therefore, it could be argued that the present findings do not apply to the wider population of typical AD. On the other hand, studying this clinical population furthers our understanding of the disease mechanisms driving heterogeneity in AD and helps reappraise the diagnostic value of standard diagnostic markers for less typical forms of AD.

The absence of an association between visual and volumetric measures of parietal lobe volume was against our predictions and could be attributed to the two methods measuring different features of the same region. As proposed by Fumagalli and colleagues, the visual rating score may reflect more the widening of a sulcus and the increase in CSF, while the volumetric analyses are more closely associated with both grey and white matter volume.^43^

Our study's limitations include the lack of a measure of disease severity, such as the Mini‐Mental State Examination. Another limitation is that visual rating scores were provided once and by a single rater. This means that we were not able to assess intra‐ and inter‐rater reliability directly. However, it is important to note that most of these scales have been found to have good intra‐ and inter‐rater reliabilities.^15^ Moreover, due to the unavailability of visual scores in the healthy control group, we were unable to formally examine the sensitivity and specificity of these scales. Finally, the power to detect differences between the three groups was limited by the relatively small sample size of the amyloid‐negative group.

In conclusion, this study has highlighted the key issues that require addressing before visual rating scales can be recommended for regular use in the diagnostic workup of AD. Consensus guidelines on the definition of VRS abnormality are necessary to determine the optimal use of these scales in clinical practice. Future studies should also validate abnormality cut‐offs for each hemisphere, which would enable better detection of unilateral or asymmetric atrophy. We also showed the importance of biomarker confirmation of AD to assess the performance of standard diagnostic examinations in the atypical AD population. Atypical forms may go undetected in studies of clinically diagnosed AD because of the lack of the typical hallmarks of the disease, with the consequent risk of a selection bias towards those with typical atrophy patterns. The intragroup heterogeneity shown by this study points to the need to characterize AD neuroimaging profiles, especially in the presence of atypical phenotypes, for the potential identification of more sensitive and specific markers. While in the medium‐long term AD diagnosis is increasingly likely to rely on biomarkers of amyloid and tau, the present work is highly relevant to current clinical practice, which still places a major focus on the visual assessment of MRI scans.

## Funding Information

The work was funded by Alzheimer's Society (grant number P75464) and supported by the NIHR Biomedical Research Centre at Imperial College London. None of the funders was involved in the conduct of the study or preparation of the article.

## Author Contributions

FL designed the study, collected and analysed the data and wrote the manuscript; AG designed the study and performed the visual ratings; NP and ZW contributed to data collection; RP conceived the idea and designed the study; PM conceived the idea, designed the study and wrote the manuscript; all authors reviewed and approved the manuscript.

## Conflict of Interest Statement

ZW previously participated in the Eli Lilly PET advisory board and was an amyloid‐PET read trainer. CC has taken part in an advisory panel for Roche Pharmaceuticals. RP previously sat on an advisory board for Eli Lilly and received support from GE for research imaging from 2014 to 2018. PM has given an educational talk at a meeting organized by GE. None of the authors currently have funding or support from any commercial organization involved in amyloid PET imaging. FL, AG, GS, and NP report not disclosures relevant to the manuscript.

## Ethics Approval

Ethical approval for this study was obtained from the Camden and Kings Cross UK Research Ethics Committee (REC number 20/LO/0442) and the Cambridge East Research Ethics Committee (REC number 10/H0304/70).

## Supporting information


Figure S1

## Data Availability

Data not provided in the article are available upon reasonable request.
