# Supplementary material for: Visual atrophy rating scales and amyloid PET status in an Alzheimer's disease clinical cohort
Source: Ann Clin Transl Neurol. 2023 Mar 5;10(4):619–31. doi: 10.1002/acn3.51749 (PMC10109315; doi:10.1002/acn3.51749)
Supplement: Supplementary file 1 — Figure S1 [file ACN3-10-619-s001.docx]

**Supplementary Fig. 1** (**A**) Comparison of mean rating scores in the AD group of the current cohort (APC Cohort, n=39) with the AD (n=101) and CN (n=73) individuals included in Harper et al’s study^15^. (**B**) Comparison of mean rating scores in the early onset amnestic (n=12) and non-amnestic (n=8) AD groups of the current cohort with the respective groups (n=48 and 22 respectively) from Falgàs et al’s study^14^

* p<.05

**Cohort comparisons**

Panel A of Figure 4 compares our AD clinical cohort (n=39) with the AD (n=101) and CN (n=73) subjects from Harper et al’s study^15^. Interestingly, our AD group’s mean scores were comparable to the CN group for most scales, even after matching the two AD groups for severity by removing MCI cases from our group. Moreover, our AD group was on average older than Harper et al’s groups, suggesting that this finding was not attributable to higher age-related atrophy in the other AD cohort. However, the MTA and PA scores were consistent across the two AD cohorts, further corroborating the relevance of these scales in patients with Alzheimer’s pathology. Panel B of Figure 4 compares our subset of EOAD patients (onset < 65 years), with amnestic (n=12) and non-amnestic (n=8) presentations, with the equivalent groups from Falgàs et al’s study^14^ (n=48 and 22 respectively). Mean scores were generally comparable between the two cohorts, with a trend towards higher MTA and PA scores in both the amnestic and non-amnestic EOAD groups of the present cohort. None of the above studies included the Fazekas or the ERICA scales.

**Supplementary Fig. 2** Comparison of visual rating scores between amnestic and non-amnestic patients in the amyloid-positive groups. Non-parametric testing revealed significant differences in the OF, PA and FAZEKAS PVH scales

* p<.05; **p<.01

**Supplementary Table 1. MTA and PA cut-offs proposed by different studies**

|  |  | Scheltens et al. 1992 ^7^ | Ferreira et al. 2015 ^11^ | Rhodius-Meester et al. 2017 ^10^ | Claus et al. 2017 ^13^ | Pereira et al. 2014 ^9^ |
| --- | --- | --- | --- | --- | --- | --- |
| **MTA** ^7^ | < 65 y | ≥ 2 | ≥ 1.5 | ≥1 | ≥1 | ≥ 1.5 |
|  | 65-74 y | ≥ 2 | ≥ 1.5 | ≥ 1.5 | ≥ 1.5 | ≥ 1.5 |
|  | 75-84 y | ≥3 | ≥ 2 | ≥ 2 | ≥ 2 | ≥ 2 |
|  | ≥ 85 y | ≥3 | ≥ 2.5 | ≥ 2 | ≥ 2 | ≥ 2 |
|  |  | Koedam et al. 2011 ^20^ | Ferreira et al. 2015 ^11^ | Rhodius-Meester et al. 2017 ^10^ |  |  |
| **PA** ^20^ | < 65 y | ≥2 | ≥1 | ≥1 |  |  |
|  | 65-74 y | ≥2 | ≥1 | ≥2 |  |  |
|  | 75-84 y | ≥2 | ≥1 | ≥2 |  |  |
|  | ≥ 85 y | ≥2 | ≥1 | ≥2 |  |  |

MTA, medial temporal lobe atrophy; PA, posterior atrophy

**Supplementary Table 2** **Review of studies examining PA and MTA in AD**

| **Author, year of publication** | *General Characteristics* | | | | |
| --- | --- | --- | --- | --- | --- |
|  | **Country** | **Study design** | **Cohort type** | **Confirmation of AD** | **Diagnostic criteria for MCI/AD** |
| Pyun et al. (2017)  ^23^ | South Korea | retrospective, longitudinal | Research (ADNI) | yes (CSF) | MMSE≥24, CDR≥0.5, WMS delayed recall score < 1.5SD |
| Lehmann et al. (2012)  ^19^ | United Kingdom | retrospective, cross-sectional | Clinical | yes (pathology and genetic) | NINCDS-ADRDA |
| Lehmann et al. (2013)  ^48^ | United Kingdom | retrospective, longitudinal | Research (ADNI) | no | NINCDS-ADRDA |
| Kim et al. (2017)  ^24^ | South Korea | retrospective, longitudinal | Clinical | no | performance on min 1of 5 neuropsychological tests < 1.5SD and preserved ADL |
| Sheng et al. (2020)  ^21^ | China | prospective, cross-sectional | Clinical | no | Cohort A: Petersen 2004  Cohort B: Jak & Bondi 2014 |
| Suh et al. (2019)  ^26^ | South Korea | retrospective, longitudinal | Clinical | no | NIA–AA |
| Zhu et al. (2021)  ^16^ | China | prospective, cross-sectional | Clinical | no | NINCDS-ADRDA |
| O'Donovan et al. (2013)  ^17^ | United Kingdom | retrospective, cross-sectional | Clinical | no | NINCDS-ADRDA |
| Rhodius-Meester et al. (2017) ^10^ | Netherlands | prospective, longitudinal | Clinical | no | NINCDS-ADRDA, NIA AA, Petersen 2004 |
| Smits et al. (2014)  ^25^ | Netherlands | prospective, cross-sectional | Clinical | no | NIA-AA |
| Ferreira et al. (2015)  ^11^ | Sweden | retrospective, longitudinal | Research (ADNI) | no | NINCDS-ADRDA, DSM IV |
| Harper et al. (2016)  ^15^ | United Kingdom | retrospective, cross-sectional | Clinical | yes (pathology) | not specified |
| Falgás et al. (2020)  ^14^ | Spain | prospective, cross-sectional | Clinical | yes (CSF) | NIA-AA |
| Yuan et al. (2019)  ^34^ | China | prospective, cross-sectional | Clinical | no | NINCDS-ADRDA |

**Supplementary Table 2** (continued 1/4)

| **Author, year of publication** | *Study Sample Characteristics* | | | | |
| --- | --- | --- | --- | --- | --- |
|  | **Patient Group *(n)*** | **Control Group *(n)*** | **Mean age  (y±SD)** | **Male:Female** | **MMSE (mean±SD)** |
| Pyun et al. (2017)  ^23^ | 258 Aβ-pos MCI | none | **Median (IQR)** 74.1 (69.5-78.5) | 157:101 | **Median (IQR)** 27 (25-29) |
| Lehmann et al. (2012)  ^19^ | 62 AD 40 FTLD | 50 HC | AD 58.2±10.6 FTLD 59.2±8.9 HC 59.7±11.3 | AD 34:28 FTLD 26:14 HC 29:21 | AD 17.2±6.8 FTLD 22.9±5.2 HC 29.1±1.1 |
| Lehmann et al. (2013)  ^48^ | 192 MCI 99 AD | 114 HC | AD 74.9±7.9 MCI 74.4±7.4 HC 75.6±5.2 | AD 58:41 MCI 129:63 HC 58:56 | AD 23.6±1.9 MCI 26.9±1.8 HC 29.1±1 |
| Kim et al. (2017)  ^24^ | 101 SMCI  47 PMCI | 46 HC | SMCI 72.1±5.93 PMCI 72.7±4.66 HC n/a | SMCI 41:60 PMCI 18:29 HC n/a | n/a |
| Sheng et al. (2020)  ^21^ | A) 73 aMCI  B) 33 aMCI | A) 48 HC B) 45 HC | A) aMCI 68.63±9.24 A) HC 65.71±6.67 B) aMCI 64.94±8.28 B) HC 63.09±4.8 | A) aMCI 33:40 A) HC 19:29 B) aMCI 17:16 B) HC 18:27 | n/a |
| Suh et al. (2019)  ^26^ | 159 AD | none | 76.4±7.7 | 68:91 | 21.3±2.95 |
| Zhu et al. (2021)  ^16^ | 173 AD 97 DLB | 30 HC | AD 69.6±9.66 DLB 72.22±7.3 HC 69.8±6.75 | AD 70:103 DLB 44:53 HC 16:14 | *Median (IQR)* AD 15 (8-19) DLB 16 (11-21) HC 29 (28-30) |
| O'Donovan et al. (2013)  ^17^ | 36 AD 35 DLB | 35 HC | AD 78.3±5.8 DLB 78.4±6.9 HC 76.7±5.2 | AD 21:14 DLB 27:8 HC 20:14 | AD 19.5±4.4 DLB 20.3±5.3 HC 28.1±1 |
| Rhodius-Meester et al. (2017) ^10^ | 681 MCI 1347 AD | 906 HC | MCI 69±9 AD 69±9 HC 62±9 | MCI 410:271 AD 631:716 HC 502:404 | MCI 26±2 AD 20±5 HC 28±2 |
| Smits et al. (2014)  ^25^ | 329 AD | none | 67±8 | 154:175 | 20±5 |
| Ferreira et al. (2015)  ^11^ | 322 AD 385 MCI-S 95 MCI-C | 345 HC | AD 75.6±7.14 MCI-S 75.08±6.89 MCI-C 74.25±6.54 HC 74.91±5.8 | AD 146:176 MCI-S 233:152 MCI-C 56:39 HC 169:176 | AD 21.53±5.4 MCI-S 27.13±1.71 MCI-C 26.55±1.81 HC 28.92±2.46 |
| Harper et al. (2016)  ^15^ | 101 AD 28 DLB 55 FTLD | 73 HC | AD 61.1±11.4 DLB 70.1±5.9 FTLD 61.1±8.8 HC 66.6±7.9 | AD 62:39 DLB 21:7 FTLD 31:24 HC 38:35 | AD 17.5±6.0 DLB 20.1±4.6 FTLD 22.7±5.9 HC n/a |
| Falgás et al. (2020)  ^14^ | 48 sA-EOAD  22 sNA-EOAD 25 Ndg-MCI 11 bvFTD 9 svPPA 5 nfvPPA  7 gFTD | 42 HC | sA-EOAD 59.5±4.2 sNA-EOAD 57.4±3.8 Ndg-MCI 59.6±4.1 bvFTD 60.9±5.2 svPPA 60±4.3 nfvPPA 61.2±3.4 gFTD 59.2±4.7 HC 58.5±3.7 | sA-EOAD 18:30 sNA-EOAD 12:10 Ndg-MCI 13:12 bvFTD 8:3 svPPA 6:3 nfvPPA 3:1 gFTD 1:6 HC 9:33 | sA-EOAD 23±3.6 sNA-EOAD 24.2±3.6 Ndg-MCI 26.4±2.5 bvFTD 24.9±3.3 svPPA 26.3±3 nfvPPA 25.8±2.5 gFTD 25.1±3.5 HC 28.7±1.2 |
| Yuan et al. (2019)  ^34^ | 100 AD | 100 HC | AD 63.44±9.97 HC 63.46±8.17 | AD 31:69 HC 35:65 | AD 15.7±6.6 HC 27.5±1.3 |

**Supplementary Table 2** (continued 2/4)

| **Author, year of publication** | *Visual Rating Protocol* | | | *Results* |
| --- | --- | --- | --- | --- |
|  | **VRS,  Raters *(n)*** | **LH & RH^a^** | **VRS Cut-offs** | **% Abnormal scores in AD/MCI** |
| Pyun et al. (2017)  ^23^ | PA, MTA  3 raters | mean | MTA ≥ 1.5 below 75y MTA ≥ 2 below 75y PA ≥ 1.5 all ages | MTA only: 12.8%  PA only: 32.6%  MTA & PA: 24.9%  None: 20% |
| Lehmann et al. (2012)  ^19^ | PA, MTA  2 raters | mean | MTA >1 all ages PA > 1 all ages | MTA only: 18% PA only: 30% MTA & PA: 34% None: 18% |
| Lehmann et al. (2013)  ^48^ | PA, MTA  1 rater | mean | n/a | not reported |
| Kim et al. (2017)  ^24^ | PA, MTA, FA  1 rater | sum | MTA ≥ 3 all ages  PA ≥ 3 all ages FA ≥ 2 all ages | MTA only: 25% PA only: 13% MTA & PA: 32% None: 29.7% |
| Sheng et al. (2020)  ^21^ | PA, MTA  3 raters | mean | not specified | not reported |
| Suh et al. (2019)  ^26^ | PA, MTA, FA  3 raters | mean | PA ≥ 1.5 all ages MTA ≥ 2 all ages FA ≥ 1 all ages | PA: 50.3% MTA: 70.4% |
| Zhu et al. (2021)  ^16^ | PA, MTA, FA, Fazekas  3 raters | n/a | not specified | not reported |
| O'Donovan et al. (2013)  ^17^ | PA, MTA, VEn  1 rater | sum | not specified | not reported |
| Rhodius-Meester et al. (2017) ^10^ | MTA, PA, GCA, Fazekas  1 rater | mean | MTA ≥ 1 below 65y MTA ≥ 1.5 65-75y  MTA ≥ 2 above 75y PA ≥ 1 below 65y PA ≥ 2 above 65 GCA ≥ 1 below 65y | Limited to EOAD: MTA only: 8% PA only: 24% MTA & PA: 29% None: 39% |
| Smits et al. (2014)  ^25^ | MTA, PA  not specified | mean | PA ≥ 1.5 all ages MTA ≥ 1.5 all ages | MTA only: 30% PA only: 17% MTA & PA: 26% None: 27% |
| Ferreira et al. (2015)  ^11^ | MTA, PA, FA  1 rater | mean | MTA ≥ 1.5 below 75y MTA ≥ 2 75-84y MTA ≥ 2.5 above 85y PA ≥ 1 all ages | not reported |
| Harper et al. (2016)  ^15^ | MTA, PA, AT, OF, FI, AC  2 raters | mean | MTA between ≥ 1.5 and ≥ 2  PA between ≥ 2.5 and ≥ 3 | not reported |
| Falgás et al. (2020)  ^14^ | MTA, PA, AT, OF, FI, AC  2 raters | mean | MTA between ≥ 0.5 and ≥ 1.5 PA between ≥ 1 and ≥ 1.5 | not reported |
| Yuan et al. (2019)  ^34^ | MTA, PA, AT, OF, FI, AC  2 raters | mean | MTA ≥ 1.5 all ages PA ≥ 1.5 all ages | not reported |

**Supplementary Table 2** (continued 3/4)

| **Author, year of publication)** | *Results (continued)* | |
| --- | --- | --- |
|  | **SE & SP or AUC (ranges)^b^** | **Author’s main conclusions** *(adapted)*^c^ |
| Pyun et al. (2017)  ^23^ | n/a | PA in Aβ-pos MCI is associated with disease progression to dementia, independent of MTA. |
| Lehmann et al. (2012)  ^19^ | MTA: AUC 0.66-0.93 PA: AUC 0.66-0.74 MTA & PA: 0.73-0.92 | Some AD patients may not receive the right diagnosis if only MTA is considered. PA helps to distinguish AD from FTLD, and EOAD from young HC. |
| Lehmann et al. (2013)  ^48^ | n/a | MTA predicts conversion to AD in late-onset aMCI, with some evidence that PA ratings are also useful. The MTA and PA may offer independent and complementary information on AD conversion risk. Widespread PA is associated with elevated tau. |
| Kim et al. (2017)  ^24^ | n/a | PA predicts MCI progression to AD independently of MTA. |
| Sheng et al. (2020)  ^21^ | MTA: AUC 0.776-.822  PA: AUC 0.725 -0.721 MTA & PA: AUC 0.818-0.824 | The combination of MTA and PA provides better diagnostic accuracy than single visual rating measures. |
| Suh et al. (2019)  ^26^ | n/a | PA but not MTA scores predict rapid cognitive progression of AD cognitive symptoms over 1 year. |
| Zhu et al. (2021)  ^16^ | MTA: SE 0.497-0.979, SP 0.633-1.00 MTA & PA & FA: SE 0.665-0.971, SP 0.742-0.933 MTA & PA & FA & Fazekas: SE 0.647-1, SP 0.804-1 | The combination of MTA, PA, FA and Fazekas scales provides better diagnostic accuracy for the differentiation between AD and DLB. |
| O'Donovan et al. (2013)  ^17^ | MTA: SE 0.54-0.89, SP 0.46-0.89 PA: SE 0.69-0.75, SP 0.31-0.46 | PA is not a reliable marker for distinguishing LOAD from HC and from DLB. MTA and VEn are useful in distinguishing AD and DLB from HC. |
| Rhodius-Meester et al. (2017) ^10^ | MTA: SE 0.82, SP 0.76 PA: SE 0.84, SP 0.71 MTA & PA (in EOAD): SE 0.9,  SP 0.94 | The predictive value of MTA is stronger in older than in younger patients. The combination of MTA and PA increases sensitivity and specificity for the differentiation of younger patients. |
| Smits et al. (2014)  ^25^ | n/a | MTA and PA are associated with different cognitive domains. MTA is worse in late- than early-onset AD, while PA is comparable between the two groups. |
| Ferreira et al. (2015)  ^11^ | *Based on recommended cut-offs:* MTA: SE 55.2-85.3, SP 75.2-91.7 PA: SE 55.2-80.0, SP 31.2-75.0 MTA & PA: SE 40-49.7, SP 82.9-86.1 | MTA showed the best diagnostic performance, followed by FA and PA. Combining MTA with FA or PA, or both, did not increase AUC values for AD identification. |
| Harper et al. (2016)  ^15^ | MTA: SE 0.64-0.82, SP 0.68-0.89 PA: SE 0.07-0.22, SP 0.86-1 | MTA is the best single scale to differentiate LOAD from DLB but not FTLD. PA is the best for accurately identifying EOAD from other groups. |
| Falgás et al. (2020)  ^14^ | MTA: SE 0.63-1, SP 0.81-0.98 PA: SE 0.68-0.86, SP 0.60-0.88 | PA is the best to differentiate NA-EOAD but AUC not > 0.80. Combining different scales improves the diagnostic accuracy for some diagnostic groups. |
| Yuan et al. (2019)  ^34^ | MTA: SE 0.62, SP 0.95 PA: SE 0.72, SP 0.56 | Each single VRS can effectively distinguish AD  from HC. |

**Supplementary Table 2** (continued 4/4)

|  |
| --- |

ADNI, Alzheimer's Disease Neuroimaging Initiative; CSF, cerebrospinal fluid; MMSE, mini-mental state examination; CDR, clinical dementia rating; WMS, Wechsler memory scale; NINCDS-ADRDA, National Institute of Neurological and Communicative Diseases and Stroke/Alzheimer's Disease and Related Disorders Association; NIA-AA, National Institutes of Health and the Alzheimer’s Association; ADL, activities of daily living; Aβ-pos, amyloid-positive; AD, Alzheimer’s disease; MCI, mild cognitive impairment; FTLD, frontotemporal lobar degeneration; HC, healthy controls; SMCI or MCI-S, stable MCI;PMCI, progressive MCI; MCI-C, MCI converters; aMCI, amnestic MCI; DLB, dementia with Lewy bodies; EOAD, early onset AD; LOAD, late-onset AD; sA-EOAD, sporadic amnestic EOAD; sNA-EOAD, sporadic non-amnestic EOAD; Ndg-MCI, non-neurodegenerative MCI, bvFTD, behavioural FTD; PPA, primary progressive aphasia; svPPA, semantic variant PPA; nfvPPA, non-fluent variant PPA; gFTD, genetic FTD; VRS, visual rating scales; LH, left hemisphere; RH, right hemisphere; MTA, medial temporal atrophy; PA, posterior atrophy; FA, frontal atrophy; VEn, ventricular enlargement; GCA, global cortical atrophy; OF, orbito-frontal; FI, fronto-insular; AC, anterior cingulate; AT, anterior temporal; SE, sensitivity; SP, specificity; AUC, area under the curve; n/a, not available

Overview of studies assessing the diagnostic and/or prognostic role of Koedam et al’s PA scale ^20^, Scheltens et al’s MTA scale ^7^, and a variable range of other VRS, in AD patients. Note that the literature search was performed in July 2022 using PubMed as the main database. The exhaustive review of studies in this field is beyond the scope of this work.

^a^Where left and right hemispheres were scored separately, some studies analysed the sum score while others the mean, as specified in this column.

^b^The reported ranges take into account all group comparisons performed within each study (e.g., AD vs HC and AD vs MCI). The aim is to broadly illustrate the best and worst diagnostic performance of PA and MTA detected by these studies. The reader should refer to the original articles for further detail.

^c^The findings and conclusions reported here are adapted from original articles to emphasise the findings on PA and MTA scales.

**Supplementary Table 3 Age-specific cut-offs adopted in this study**

|  | **<65 years** | **65-74 years** | **>75 years** |
| --- | --- | --- | --- |
| MTA ^10, 13^ | ≥1 | ≥1.5 | ≥2 |
| PA ^10, 13^ | ≥1 | ≥2 | ≥2 |
| OF | ≥1 | ≥1.5 | ≥2 |
| AC | ≥1 | ≥1.5 | ≥2 |
| AT | ≥1 | ≥1.5 | ≥2 |
| FI | ≥1 | ≥1.5 | ≥2 |
| ERICA | ≥1 | ≥1.5 | ≥2 |
| Fazekas ^10, 13^ | ≥1 | ≥2 | ≥3 |
